# Supplementary material for: Modeling the evolution of COVID-19 via compartmental and particle-based approaches: Application to the Cyprus case
Source: PLoS One. 2021 May 6;16(5):e0250709. doi: 10.1371/journal.pone.0250709 (PMC8101925; doi:10.1371/journal.pone.0250709)
Supplement: Attachment — Submitted filename: report_PONE-D-20-29820.pdf [file pone.0250709.s001.pdf]

Reviewer report on “Modeling the evolution of COVID-19” PONE-D-20-29820 by C Alexandrou et al

The authors analyze the SARS-CoV-2 infection course in Cyprus using two models: a compartmental model and a particle model.
After fitting the model parameters using recorded infections up to July 15, they provide forecasts for the future.
Their forecasts proved completely wrong with respect to the actual evolution of the epidemic.
Already at the time of submission of the paper the cumulative numbers of infection had exceeded the upper bound of what the authors report in the figures of the paper. The inconsistency between their expectation and reality can be appreciated by looking at the reported infection and the range considered by the authors.

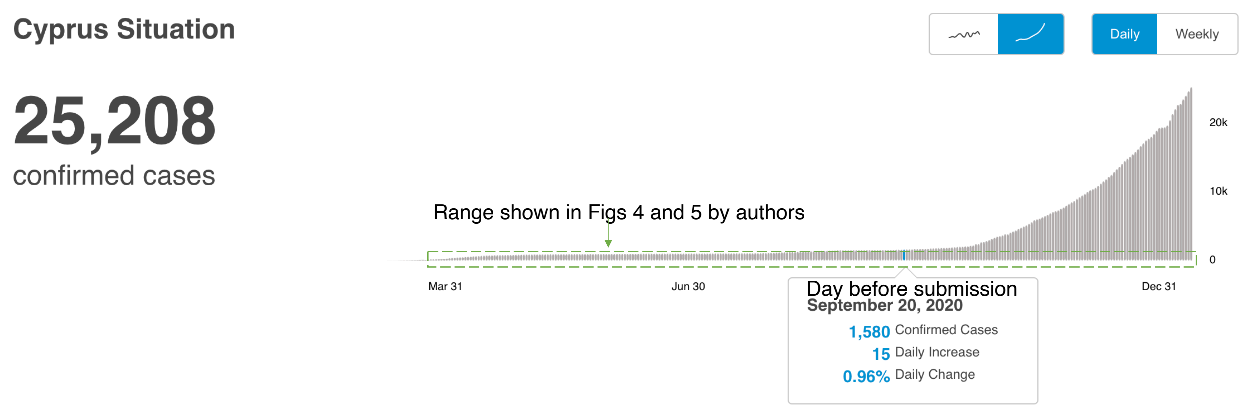

Data from WHO on the SARS-CoV2 cumulative confirmed infection cases in Cyprus retrieved on January 6 2021 from https://covid19.who.int/region/euro/country/cy shown together with a rough estimate of the range considered by the authors in the submitted manuscript (green dotted line).

The current cumulative number of cases is almost 20-times larger than the range the upper limit of the axis shown by the authors (25000 vs ~1400).
This clearly shows how the authors’ analysis is not sufficiently careful and that, with the data available at the moment of submission (and most likely now too) their methods and fitting should absolutely not be used to make forecasts. This fact alone is sufficient to recommend the rejection of the paper.
Below, you will find specific comments concerning the modeling approach and other remarks.

COMMENTS

1. The title is far too general. There are already too many papers modeling the evolution of COVID19 to use a title like this. The authors should to specify already in the title the novel/different aspects of their modeling/analysis and that they are applying it to study data from Cyprus.

2. The choice of a step-wise probability of recovery P differs, as the authors mention, from the exponentially decaying profile usually used for compartmental model like the classic SIR one. The author should motivate more clearly why they think the step-like profile is more realistic for the SARS-CoV-2 pandemic than the exponential profile. They should also discuss how much their results and predictions depend on this specific choice and what qualitative difference they expect from the usually employed exponential choice.

3. The authors allow the transmission rate to vary in time and characterize this by the use of 5 parameters. As common in similar approaches, there is some arbitrariness in how the time dependence of transmission rate is chosen. It is then difficult to make reliable forecasts based on these highly simplified models with several assumptions on the transmission rates that are hard to properly justify. Resorting to publicly available mobility data can guide the modeling, even though, it remains difficult to capture seasonality effects and the influence of other containment measures not based on reducing mobility (such as face-mask wearing). The authors should comment on this.

4. The time evolution of the fraction of detected cases r, defined in Eq. (18), is a crucial parameter in the present model. In contrast to its importance, its behavior is mostly “guessed” by the authors. The estimation of this evolution should be based on available data such as the time course of the number of tests performed and the positive rate.

5. Several recent studies have highlighted the importance of population heterogeneity (with respect to susceptibility or infectivity) for the spread of epidemics, in general and COVID19 in particular [see e.g. T. Britton, F. Ball, P. Trapman, Science *369*(6505), 846-849 2020. Gomes, M. Gabriela M., et al. "Individual variation in susceptibility or exposure to SARS-CoV-2 lowers the herd immunity threshold." *medRxiv* (2020). J. Neipelet al PLoS One, 2020, 15, e0239678. A. V Tkachenko et al arXiv2008.08142, (2020). G. N. Wong et al Phys. Rev. X 10, 041033 (2020), A. Montalbán, et al arXiv:2008.00098 (2020).] I would invite the authors to take this aspect into account or to motivate in the paper why they do not think it is relevant for their analysis.

6. I do not find the fact that it is possible to choose parameters for the particle model that match the behavior of the compartmental model to be very insightful. What knowledge is gained by performing the particle simulations?

7. Why do the predictions for Scenario A vary so much between the particle and the compartmental model? What number of cumulative cases are forecast for Scenario A in the compartmental model for the end of 2020?

MINOR COMMENTS

1. In equations (2) I would suggest not to use the variable x since it may suggest a spatial variable, which is not considered in the present model.

2. “Scenaria” is an uncommon choice for plural of scenario

3. In the plots where r(t) is plotted it would be interesting to see β(t) as well

4. One of the data links is broken: https://www.pio.gov.cy/coronavirus/en/info.html
