# Supplementary material for: Modeling the evolution of COVID-19 via compartmental and particle-based approaches: Application to the Cyprus case
Source: PLoS One. 2021 May 6;16(5):e0250709. doi: 10.1371/journal.pone.0250709 (PMC8101925; doi:10.1371/journal.pone.0250709)
Supplement: Attachment — Submitted filename: ahiks.pdf [file pone.0250709.s002.pdf]

Referee's report on the manuscript "Modeling the evolution of COVID-19" by Constantia Alexandrou, Vangelis Harmandaris, Anastasios Irakleous, Giannis Koutsou, Nikos Savva

In the paper under review, the authors give a detailed study of two models: first is the modification of classical deterministic SIR model for epidemics in the case when the transition rates between the corresponding compartments are incorporated. Second model is a particle model, in which the disease is transmitted through particle collisions and infection rates are varied by adjusting the particle velocities. Both models are applied to Cyprus data.

It is needless to say that there are thousands of papers and approaches for COVID modelling and, clearly, it is almost impossible to choose which is the right one. The authors decided to choose the simple models with a minimal number of parameters, what is rather well motivated in the case of small countries such as Cyprus. However, in general, the long-term COVID forecasting based on a mathematical model (especially deterministic SIR-type) is very questionable (see [1], [2]), especially when making the assumptions of the model.

In the context of many COVID modelling approaches, the subject and results of the paper are interesting enough, the paper is well written and I recommend it for publishing in the journal. Below, please find a list of comments.

Comments:

1. In my opinion, the title of the paper is too general and even ambitious. Please be more concrete on what is done in the paper.

2. There are many models where recovered and died are modelled separately. Please explain why these classes are not differentiated in your research (item **v** on p. 3).

3. P. 6, l. 3: c_1, c_2; line below eq. (8): b_0, b_1, b_2 and m_1, m_2, etc.

4. P. 6, eq. (7). Please recall the definition of hyperbolic tangent tanh. Also, specify the role of parameters m_j.

5. P. 8. The equalities in eqs. (14)–(16) are not very clear. Could you please explain how these expressions for effective (?) R_0 are obtained? Especially, the form $R_0^{\text{model}}(t) = \beta(t)(1 - r(t))\tau_i$.

6. The Reference list is not accurate. Many references miss the citation data (see 1, refs. 2, 5, 9, 10, 14).

Literature

Ioannidis J.P.A., Cripps S., Tanner M.A. (2020) Forecasting for COVID-19 has failed. International Journal of Forecasting. 2020. https://doi.org/10.1016/j.ijforecast.2020.08.004.

Saltelli et al. (2020) Five ways to ensure that models serve society: a manifesto. Nature. 582, 482–484.
