# Supplementary material for: Modeling the evolution of COVID-19 via compartmental and particle-based approaches: Application to the Cyprus case
Source: PLoS One. 2021 May 6;16(5):e0250709. doi: 10.1371/journal.pone.0250709 (PMC8101925; doi:10.1371/journal.pone.0250709)
Supplement: Attachment — Submitted filename: response.pdf [file pone.0250709.s003.pdf]

PONE-D-20-29820 "*Modeling the evolution of COVID-19*"

Response to reviewers' comments

We would like to thank both reviewers for their careful reading of our paper and for the feedback they have provided. We have considered all points raised and have modified the manuscript accordingly. We summarize the changes carried out in response to each of the reviewers' comments.

For better context of the time-line of our submission, we would like to point out that this manuscript was submitted on August 17th, 2020. A preprint was posted to the arXiv on August 7th, see `arXiv:2008.03165` `https://arxiv.org/pdf/2008.03165.pdf`. Therefore, only data known until mid July were used. More importantly, the models took into account the known measures and circumstances until then. It is known that such models need to be adapted if circumstances change.

Reviewer #1

- *The authors analyze the SARS-CoV-2 infection course in Cyprus using two models: a compartmental model and a particle model. After fitting the model parameters using recorded infections up to July 15, they provide forecasts for the future. Their forecasts proved completely wrong with respect to the actual evolution of the epidemic. Already at the time of submission of the paper the cumulative numbers of infection had exceeded the upper bound of what the authors report in the figures of the paper. The inconsistency between their expectation and reality can be appreciated by looking at the reported infection and the range considered by the authors. Data from WHO on the SARS-CoV2 cumulative confirmed infection cases in Cyprus retrieved on January 6 2021 from https://covid19.who.int/region/euro/country/cy shown together with a rough estimate of the range considered by the authors in the submitted manuscript (green dotted line). [...] This clearly shows how the authors' analysis is not sufficiently careful and that, with the data available at the moment of submission (and most likely now too) their methods and fitting should absolutely not be used to make forecasts. This fact alone is sufficient to recommend the rejection of the paper.*

Response: As already pointed out, our manuscript was submitted on August 17th, 2020. As is known, these models need to be informed when circumstances change, e.g. imported cases in the summer, traveling during vacations, and new measures. Therefore, the purpose of this work is to show that these models, *once taking into account known data and measures* can predict the evolution *given certain assumptions* of how the infection rate evolves beyond the point to which we fit. In a volatile situation, the forecast accuracy and the length of the forecast horizon diminishes, but they can still be reliably used for shorter term forecasts. This is precisely why we consider four different scenarios to demonstrate how these models can predict the confirmed cases given four different circumstances that combine how the infection and detection rates change and how measures are taken on subgroups of the population.

We have made several changes in the text to highlight this better. In particular, we have:

- changed the discussion on page 2, following the introduction of the two models;
- added text to the first sentence of the subsection titled "Forecasting for various scenarios", to clarify what the scenario assumptions entail;
- added text to the first paragraph of section "Results".

We believe these changes convey more clearly that our model forecasts are a tool to assess the expected number of confirmed cases under specific input i.e. given how the infection rate evolves and how certain measures are implemented, and are not to be misinterpreted as absolute predictions. These models can not predict how interactions among the population may change by e.g. imported cases from travelers nor if isolated events lead to sudden increases in the infected, which can have particularly large effects on relatively small populations like Cyprus. Including such effects is clearly beyond the scope of these models as it relies in effectively predicting changes in public policy and societal response and adherence to measures. However, the models are flexible enough to accommodate new circumstances and measures and predict the long-term time evolution *under certain assumptions* or what we call in this work *scenarios*. This is very valuable since they can serve as guidelines on the effectiveness of measures and how they are lifted to control the

spread. We demonstrate this by including an addendum at the end of the manuscript and a new figure, Fig. 6, where we fit the data up to Nov. 30th and predict until the end of the year. These predictions are in agreement with what has been observed, precisely because the epidemiological conditions in Cyprus underwent little change during that time. It also demonstrates that, with a limited set of parameters, we can model the evolution of the confirmed cases, with multiple lock-down phases and of different magnitude.

1. *The title is far too general. There are already too many papers modeling the evolution of COVID19 to use a title like this. The authors should to specify already in the title the novel/different aspects of their modeling/analysis and that they are applying it to study data from Cyprus.*

Response: We have changed the title to: "*Modeling the evolution of COVID-19 via compartmental and particle-based approaches: application to the Cyprus case*"

2. *The choice of a step-wise probability of recovery P differs, as the authors mention, from the exponentially decaying profile usually used for compartmental model like the classic SIR one. The authors should motivate more clearly why they think the step-like profile is more realistic for the SARS-CoV-2 pandemic than the exponential profile. They should also discuss how much their results and predictions depend on this specific choice and what qualitative difference they expect from the usually employed exponential choice.*

Response: We do not anticipate any qualitatively different results when using an exponentially decaying recovery probability. The consequence of exponentially distributed waiting times is indeed manifested in the tails of the curves, whereby, once the disease is about to be eradicated, the infective and quarantined groups do not diminish as fast as new infections do. For this reason, such exponentially distributed times have been criticized as being epidemiologically unrealistic. This is demonstrated in Fig. 1 of Ref. [19] as cited in our manuscript. There it is shown that constant waiting times produce a steeper increase in prevalence, reaching a peak that is significantly higher, but also an epidemic of shorter duration compared to the exponentially distributed waiting times. The authors there argue that exponentially distributed times produce "overoptimistic predictions about the low levels of control required to subdue an epidemic". Beyond this motivation, our choice to keep the waiting times τ_i and τ_e constant also allows us to capture the discrete nature of data reporting (e.g. recovered cases are officially registered when an individual tests negative a fixed number of days after onset of symptoms), whilst avoiding introducing additional parameters to describe a largely unknown probability distribution of the actual waiting times.

We have added some text before Eqs. (3) to make our choice clearer.

3. *The authors allow the transmission rate to vary in time and characterize this by the use of 5 parameters. As common in similar approaches, there is some arbitrariness in how the time dependence of transmission rate is chosen. It is then difficult to make reliable forecasts based on these highly simplified models with several assumptions on the transmission rates that are hard to properly justify. Resorting to publicly available mobility data can guide the modeling, even though, it remains difficult to capture seasonality effects and the influence of other containment measures not based on reducing mobility (such as face-mask wearing). The authors should comment on this.*

Response: We thank the referee for pointing this out. We agree with the comment and this is why we choose the specific form for $\beta(t)$ (and the velocities $v(t)$ for the particle model). While relying on few parameters, this form is quite expressive, as also shown in the new Fig. 6 included in the updated manuscript, that captures multiple changes in infection rate, both abrupt and smooth. Since mobility data are not available for Cyprus, we make relatively simple assumptions for the forecasts, e.g. by keeping $\beta(t)$ constant in the forecasting window (scenario A), or decreasing $\beta(t)$ due to new measures (scenario C), etc.

In any case, we believe certain simplifications are necessary even when incorporating additional data such as those indicated by the reviewer, given the complexity of the system and its dependence on a multitude of factors, ranging from social interactions and population movement, to health care system characteristics, population access to health care etc.

We have added text following Eq. (7) explaining in more detail our choice of this form of $\beta(t)$.

4. *The time evolution of the fraction of detected cases r, defined in Eq. (18), is a crucial parameter in the present model. In contrast to its importance, its behavior is mostly "guessed" by the authors. The estimation of this evolution should be based on available data such as the time course of the number of tests performed and the positive rate.*

Response: In fact, we have found that in practice the quality of the fits is fairly robust against small changes in the initial and final detection rate, provided we exclude cases at the two extremes, i.e. $r(t) \simeq 0$ or $r(t) \simeq 1$. This is likely because we rely on the confirmed cases, which are the only data available on a systematic basis, and the factor containing r can be absorbed within $\beta(t)$ by a corresponding rescaling of the undetected cases. As for the time chosen for this transition, we show the evolution of daily tests and positivity in Fig. 1 of this report. As can be seen, the

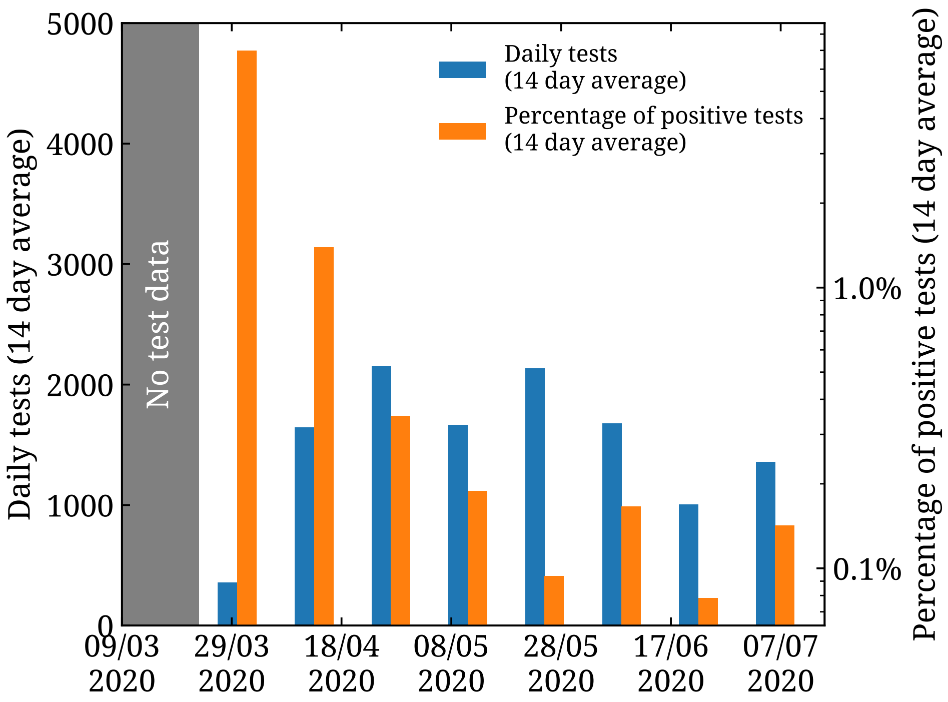

Figure 1: 14-day average of the daily tests (blue bars) and of the percentage of daily tests returned positive (orange bars) for Cyprus. The former is plotted on the left vertical axis while the latter on the right vertical axis.

positivity rate and average daily tests stabilize around May, the former to about 0.1% and the latter to around 2000 PCR tests. This motivates placing the transition of $r(t)$ from 0.9 to 0.7 within May. We add some details regarding this in the text that follows Eq. (18).

5. *Several recent studies have highlighted the importance of population heterogeneity (with respect to susceptibility or infectivity) for the spread of epidemics, in general and COVID19 in particular [see e.g. T. Britton, F. Ball, P. Trapman, Science 369 (6505), 846-849 2020. Gomes, M. Gbriela M., et al. "Individual variation in susceptibility or exposure to SARS-CoV-2 lowers the herd immunity threshold." medRxiv (2020). J. Neipel et al. PLoS One, 2020, 15, e0239678. A. V Tkachenko et al. arXiv2008.08142, (2020). G. N. Wong et al., Phys. Rev. X 10, 041033 (2020), A. Montalbàn, et al. arXiv:2008.00098 (2020). I would invite the authors to take this aspect into account or to motivate in the paper why they do not think it is relevant for their analysis.*

Response: As explained in our paper, our models allow for modeling subgroups with different transmission rates between them. With the availability of data such as those described by the reviewer, these data can be taken into account when fitting. However, to our knowledge these are not available for the case of Cyprus, at least not in a form and volume that can be used during modelling and parameter fitting. We demonstrate how this feature of our models can be applied in forecasting (scenario D), and comment on this adding a paragraph to the subsection titled "Forecasting for various scenarios".

We also note that the work quoted by the reviewer appeared after we finalized and submitted this work.

6. *I do not find the fact that it is possible to choose parameters for the particle model that match the behavior of the compartmental model to be very insightful. What knowledge is gained by performing the particle simulations.*

Response: The parameters of the particle model are chosen to match the behavior of the data, not of the compartmental model. The fact that they agree in their prediction for a given scenario is insightful, since the errors quoted for the two models reflect different sources of uncertainty. We clarify this in the paragraph that follows the introduction of the two models on page 2.

7. *Why do the predictions for Scenario A vary so much between the particle and the compartmental model? What number of cumulative cases are forecast for Scenario A in the compartmental model for the end of 2020?*

Response: For the compartmental model, the number of predicted cumulative cases for the 31st of Dec. has central value 2302 with 90% confidence interval yielding the range 1101 – 3556. For the particle model, the same forecast is 1352 with range 1099 – 1674. The forecasted cumulative cases, therefore, agree within errors for the two models.

The different ranges are to be expected, given that the two models are rather different; the compartmental model is deterministic, unlike the particle model which is stochastic. In the newly provided Fig. 6, the end-of-the-year forecasts lie between 22,060 and 28,200 for the compartmental model and 7389 and 28,186 for the particle model, while the actual reported cases where 22,651 on Dec. 31st 2020.

Minor comments

1. *In equations (2) I would suggest not to use the variable x since it may suggest a spatial variable, which is not considered in the present model*

Response: We have changed Eqs. (2) to use τ as the integration variable.

2. *"Scenaria" is an uncommon choice for plural of scenario*

Response: We now use "scenarios" as the plural of "scenario", throughout

3. *In the plots where $r(t)$ is plotted it would be interesting to see $\beta(t)$ as well*

Response: We have added $\beta(t)$ to those plots

4. *One of the data links is broken:* `https://www.pio.gov.cy/coronavirus/en/info.html`

Response: We have updated the link.

Reviewer #2

1. *In my opinion, the title of the paper is too general and even ambitious. Please be more concrete on what is done in the paper.*

Response: As in response to Reviewer #1, the title is now changed to: "*Modeling the evolution of COVID-19 via compartmental and particle-based approaches: application to the Cyprus case*"

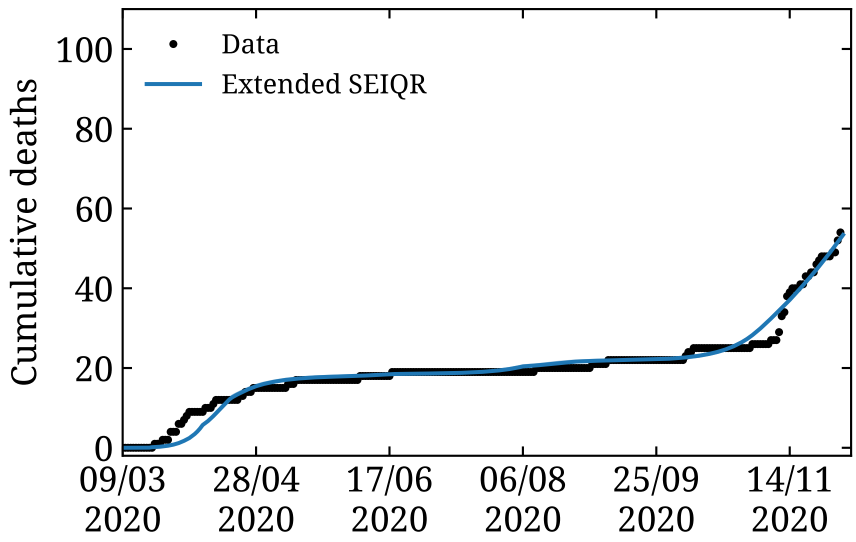

Figure 2: Reported deaths in Cyprus related to COVID-19 (black circles) and modeled deaths based on our extended SEIQR model.

2. *There are many models where recovered and died are modelled separately. Please explain why these classes are not differentiated in your research (item v on p. 3).*

Response: We can model deceased as a percentage of the recovered provided enough data for the death rate are available and there is a statistically significant number of deaths. As explained in our paper, we are largely motivated in developing models that use confirmed cases alone, suitable for populations where number of deaths are low. Indeed for Cyprus, the number of deaths, especially during the initial phase of the pandemic, are too low and do not allow for a robust statistical analysis when separating recovered from deceased. While modeling the deaths is beyond the intended scope of this work, we provide in Fig. 2 of this reply a preliminary analysis using our extended SEIQR model to model deaths up to the end of November. As can be seen the modeling is more reliable as the number of deaths increases. We have modified item v on page 3 commenting on this.

3. *P. 6, l. 3: c1, c2; line below eq. (8): b0, b1, b2 and m1, m2, etc.*

Response: Changes made, including a number of occurrences elsewhere, e.g. those of $r_{1,2}(t)$, $N_{1,2}$, etc.

4. *P. 6, eq. (7). Please recall the definition of hyperbolic tangent tanh. Also, specify the role of parameters* m_j.

Response: We have added this in the discussion following Eq. (7).

5. *P. 8. The equalities in eqs. (14)–(16) are not very clear. Could you please explain how these expressions for effective (?)* R_0 *are obtained? Especially, the form* $R_0^{model}(t) = \beta(t)(1 - r(t))\tau_i$.

Response: We have elaborated on the definitions of the effective reproduction number in Sec. 2.3.

We now indicate the effective reproduction number with $R(t)$ throughout the paper and reserve R_0 to be the basic reproduction number $R_0 = R(0)$. This change is included in the figures.

6. The Reference list is not accurate. Many references miss the citation data (see 1, refs. 2, 5, 9, 10, 14).

Response: We have fixed Ref. [1] and have added the missing data for Refs. [5, 9, 10, 14]. Ref. [2] follows the citation as indicated at `https://ourworldindata.org/coronavirus`

Additional changes

- We have added a reference to the daily updated dataset for COVID-19 published by the Cyprus government on its official data repository, Ref. [32] of the current version.
- We have added a term that was missing from Eq. (4e). This omission was not carried over to Eq. (5e) which is correct, nor to any of our implementations, and therefore does not affect any of our results.
