# Supplementary material for: Modeling the evolution of COVID-19 via compartmental and particle-based approaches: Application to the Cyprus case
Source: PLoS One. 2021 May 6;16(5):e0250709. doi: 10.1371/journal.pone.0250709 (PMC8101925; doi:10.1371/journal.pone.0250709)
Supplement: Attachment — Submitted filename: response.pdf [file pone.0250709.s004.pdf]

PONE-D-20-29820 "*Modeling the evolution of COVID-19 via compartmental and particle-based approaches: application to the Cyprus case*"

Response to reviewer's comments

We thank the reviewer for considering our changes and for their positive response to the updated manuscript. Below are our replies to each comment.

Main comments

1. *Limitations to the forecasting potential of the model.*
In my previous report this was the main criticism to the manuscript. The authors have partially addressed it but mostly in their reply. They must state the limitations more clearly and make them more evident in the manuscript. For instance, concerning the statement:
"This study also demonstrates the success of these models when relatively small numbers of cases are involved and for which statistical models yield results with very large uncertainties." I find it difficult to claim the modeling was successful. The authors must state that the predictions are reliable only for short period of times and only if the future evolution of infection rates and testing rates is specified in detail.
I still find the long-term predictions shown in the plots to be too far fetched and potentially misleading if employed for policy-making.

Response: We have revised the sentence quoted by the reviewer in the updated manuscript. Furthermore, we would like to point out the text from the original manuscript on page 2: "The main goal of the study is to examine the forecasting potential of the above models for the short-term evolution of COVID-19, under various conditions related to imposing or lifting measures." This statement is in agreement with the referee's comment. Furthermore we caution our longer term predictions with an additional paragraph on page 14, following the introduction of the four scenarios. This addition also states that our scenarios are idealizations, as they assume smooth changes of the infection rate over long periods of time.

We still maintain that the usefulness and utility of our work is that it may be used as a *decision-support tool*, when contemplating on the expected efficacy of measures in force if these are maintained in the future (assumed to be equivalent to maintaining $\beta(t)$ constant). We have shown, in Fig. 3 for example, as well as in the addendum, that the model we have utilized can be used for short-term forecasts keeping in mind the arguably strong assumption that the way the population collectively behaves remains the same in the future (i.e. maintaining the same value for $\beta(t)$ in the forecasting window).

In order to give an intuitive measure of how restrictive the limitations of the models are, the authors should show, together with the predictions, the actual development of the confirmed case. This additional information would give a clear representation of how severe the limitations are and make it easier to appreciate that the value of these kinds of models is mostly on short term predictions.
The actual time evolution of the confirmed cases must be shown in all panels of Figure 4, 5 and 6. If the plots become difficult to read because of the difference in magnitude between predictions and reality the authors can provide additional figures. However, I strongly recommend that in each figure both predictions and confirmed cases are always shown.

Response: In Fig. 8 (formerly Fig. 6) we plot scenario A on the same plot with the long term prediction. This clearly shows that the example scenarios assumed in Figs. 6 and 7 (formerly Figs. 4 and 5) did not materialize, and therefore the evolution of the pandemic did not follow the predictions of neither of the four scenarios. We have added a paragraph to explain this further, following the discussion of these two figures, before the conclusions.

We have also added the Cyprus data up until the end of 2020 in Fig. 8, following the referee's recommendation. In Figs. 6 and 7 we present our results given the situation at the time of writing.

2. *The authors state "We stress that additional scenarios can be analyzed to reflect new different circumstances as they evolve e.g. an increase of cases from incoming people, etc. The strengths of the models is that they can be adjusted to new measures and human behavior by adjusting the reproduction number. We include an addendum with an example of how these measures can be included using data that have become available after submission of this work."*
   I would recommend adding a statement to stress that this is not completely trivial and that it can become difficult to interpret what the new evolutions of the reproduction number mean. For instance, is there a possible interpretation for these 7 step-like changes that are used for fitting the additional data? How would one account for smooth changes in the reproductive number such as the ones usually due to seasonality? The authors must comment on this in the manuscript

   Response: We have added such a statement after the text quoted by the referee. We have also added text to the addendum regarding the inflection points.

3. *The authors state "We find our fits are robust to small changes r0 and r1 as long as their ratio is maintained and excluding extrema such as ..." It would be good to see a more quantitative assessment of this statement, perhaps plotting what changes in the predictions using different choices of r0 and r1.*

   Response: We have added a new figure (Fig. 5) that compares different choices of r_0 and r_1.

4. *The additional plots provided by the authors in reply to my former question (4) and parts of the authors' reply should be included in the manuscript. This would make it easier to quantify their statements*

   Response: The figure has been included as Fig. 4, including text from our response.

5. *The reply to my former question (7) contains useful information, it should be added in the main text. Also, more importantly, the plots should not cut the values of scenario A for the compartmental model. The use of insets may help the author provide all the necessary information.*

   Response: The figure ranges have been adjusted. The values quoted in the reply have been included in the manuscript.

Other comments

1. *The authors introduced a remark that is useful to better appreciate the value of the their two, complementary, modeling approaches. However, the new statement "This leads to confidence on the model forecasts on one hand, but also allows us to attribute different errors to the predictions, since the particle model exhibits stochastic errors, related to how the pandemic was seeded within the population, while the compartmental model exhibits modeling uncertainty, based on the uncertainties of the fitted parameters." requires some clarification. It seems like the stochastic models require parameter fitting as well. It would be more precise to say that their uncertainty is due both to stochasticity and uncertainty in parameters.*

   Response: We have adjusted the sentence accordingly.

Additional changes

We have added a new reference (Ref. [29] in the new manuscript) which links to an online platform that we now maintain in which fits and predictions using our extended SEIQR model can be evaluated online in real-time.
